# The complete chloroplast genome sequence of *Quercus chungii* (Fagaceae)

**DOI:** 10.1080/23802359.2021.1931505

**Published:** 2021-05-31

**Authors:** Xiao-Long Jiang, Hong-Lin Mou, Chang-Sha Luo, Gang-Biao Xu

**Affiliations:** The Laboratory of Forestry Genetics, Central South University of Forestry and Technology, Changsha, China

**Keywords:** Chloroplast genome, Fagaceae, *Quercus chungii*, ring-cup oak

## Abstract

*Quercus chungii* F.P.Metcalf, a rare oak with endemic to southern China, belongs to the compound trichome base (CTB) lineage in the *Cyclobalanopsis* section. The complete chloroplast genome of the species was assembled and annotated in this study. The circular genome was 160,731 bp in size, presenting a typical quadripartite structure including one large single-copy region (LSC, 90,140 bp), one small single-copy region (SSC, 18,911 bp), and two copies of inverted repeat regions (IRs, 25,840 bp). It encoded a total of 113 unique genes, including 79 protein-coding genes, 30 tRNA genes, and four rRNA genes. The maximum-likelihood (ML) phylogenetic tree reconstructed by IQ-TREE indicated that *Q. chungii* was more closely related to *Q. myrsinifolia* and *Q. sichourensis*.

*Quercus chungii* is a rare and precious tree that is distributed in southern China at elevations ranging from 200 to 800 m. The species belongs to compound trichome base (CTB) lineage in the *Quercus* section *Cyclobalanopsis* (Deng et al. 2018). With the rapid changes in climate and intensification of human activities, the distribution of *Q. chungii* is rapidly reduced in recent decades. Understanding the spatial genetic pattern and demographic dynamics of the species can provide important guidelines for the protection and utilization of *Q. chungii*. The genetic diversity of the species was detected by nuclear simple sequence repeats (SSRs) marker in the previous report (Jiang et al. 2019); the spatial genetic pattern and demographic dynamics of *Q. chungii* from chloroplast DNA remain largely unknown. In this study, we sequenced, assembled, and annotated the complete chloroplast genome of *Q. chungii*, it could provide useful genomic resources for the future studies on demographic dynamics of the species, and the phylogeny of ring-cupped oaks.

The sample was collected from a wild individual in the Jinpen Mountain National Forest Park, Jiangxi Province, China (25°12′36″N, 115°12′36″E, 566 m). The voucher specimens were deposited in the Herbarium of Shanghai Chenshan Botanical Garden (CSH, http://csh.ibiodiversity.net/default.html, Bin-Jie GE, gebinjie123@163.com, under the voucher number DM12068). Total genomic DNA of sample was extracted from silica-dried leaves using DNeasy plant tissue kit (TIANGEN Biotech Co., Ltd., Beijing, China). Whole genome sequencing was conducted with the Illumina Hiseq X Ten platform. A total of 66,910,243 clean reads were produced and 50,000,000 reads were used for the *de novo* assembly with GetOrganelle v1.7.2beta (Jin et al. 2020). Gene annotation was performed by the pipeline PGA (Qu et al. 2019).

The complete plastid genome of *Q*. *chungii* was a circular molecule of 160,731 bp in length. It had a typical quadripartite structure including one large single-copy (LSC) region (90,140 bp), one small single-copy (SSC) region (18,911 bp), and two copies of inverted repeat (IRs) regions (25,840 bp). The overall GC content was 36.91%, while the corresponding values of the LSC, SSC, and IR regions were 34.76%, 31.11%, and 42.77%, respectively. A total of 130 genes were encoded, of which 113 were unique and 17 were duplicated in the IR regions. Among the unique genes, 79 were protein-coding genes, 30 were tRNA genes, and four were rRNA genes.

To identify the phylogenetic position of *Q*. *chungii*, a maximum-likelihood (ML) tree was reconstructed based on the chloroplast genome sequences of *Q. chungii* and relatives species. Two species, *Trigonobalanus doichangensis* and *Fagus crenata*, were selected as outgroups. The sequences were aligned by MAFFT 7.475 (Rozewicki et al. 2019). The ML analyses were performed with IQ-TREE 1.6.12 (Chernomor et al. 2016). Node support was assessed by 1000 fast bootstrap replicates. Our result indicated that *Q*. *chungii* was more closely related to *Q*. *myrsinifolia* and *Q*. *sichourensis* with 83% bootstrap support (Figure 1).

**Figure 1.**
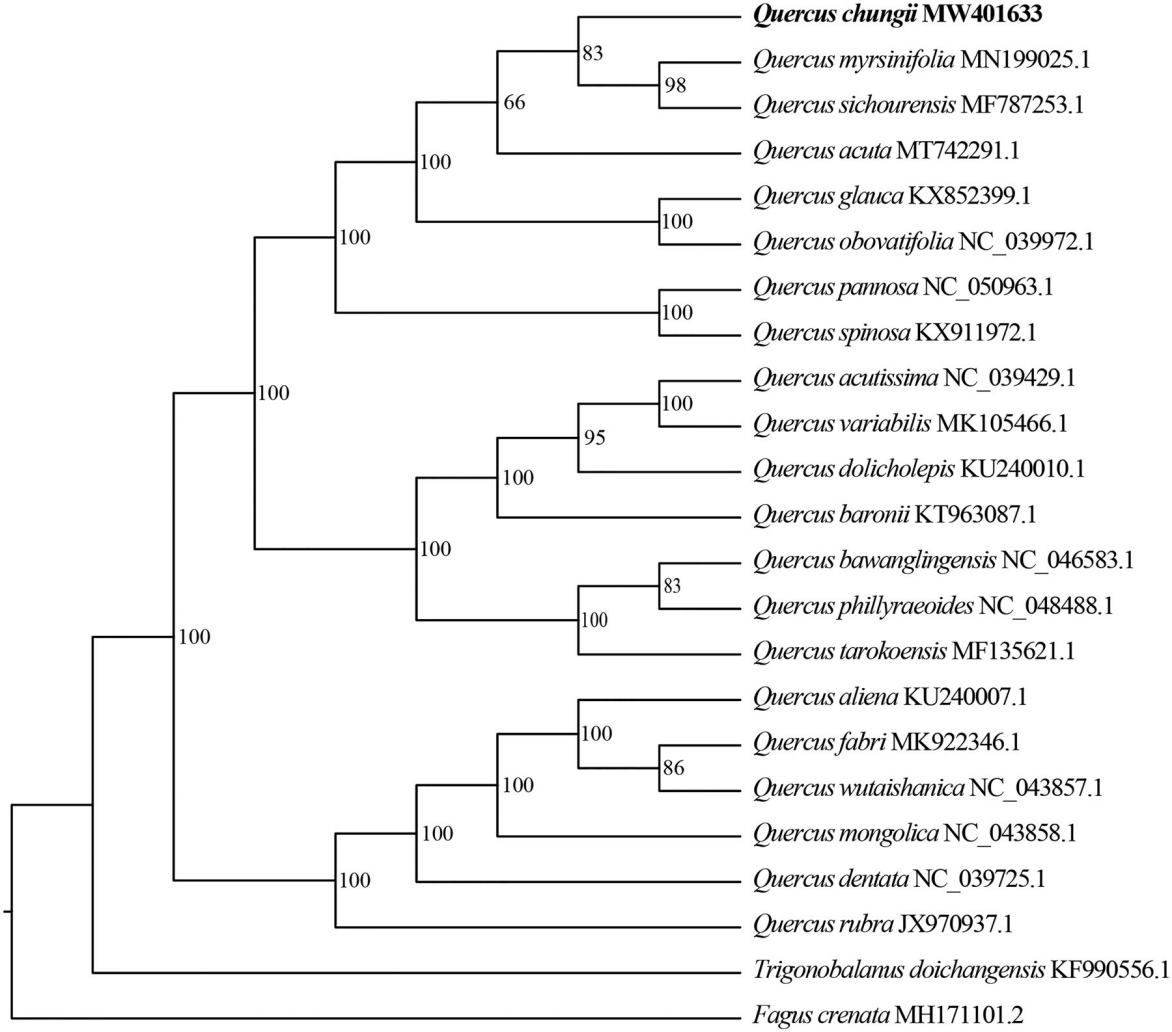
The maximum-likelihood (ML) phylogenetic tree of *Quercus chungii* and 19 relative species were reconstructed by IQ-TREE based on complete chloroplast genome sequences. The bootstrap support value is labeled for each node.

## Data Availability

The complete chloroplast genome sequence of *Quercus chungii* is deposited in the GenBank database under the accession number MW401633 (https://www.ncbi.nlm.nih.gov/nuccore/MW401633). Raw sequencing reads used in this study were deposited in the public repository BioSample with accession number SAMN18499615 (https://www.ncbi.nlm.nih.gov/biosample/SAMN18499615).
